# The effect of city reputation on Chinese corporate risk-taking

**DOI:** 10.1038/s41598-024-58922-x

**Published:** 2024-04-10

**Authors:** Sen Li, Haifeng Jiang

**Affiliations:** 1 School of Finance, Southwestern University of Finance and Economics, Chengdu, China; 2 School of Business, Fuyang Normal University, Fuyang, China

**Keywords:** National civilized city, Corporate risk-taking, City reputation, Benefit hypothesis, Scientific data, Statistics

## Abstract

City reputation is a valuable asset for the local economy and firms in the contemporary society. However, the impact of city reputation on micro-level firms has been largely overlooked by the literature. This paper uses the National Civilized City (NCC) policy in China as a quasi-natural experiment to enhance city reputation. We employ the DID approach to investigate the relationship between city reputation and corporate risk-taking. The result shows that corporate risk-taking significantly increases following the NCC policy adoption. Moreover, information asymmetry can strengthen the positive impact of city reputation on corporate risk-taking. Channel tests show that city reputation improves financial condition and decreases default risk, leading to improved risk-taking tolerance. Overall, our paper indicates that city reputation is an important mechanism to improve corporate financial performance, providing empirical evidence for local governments to pursue the NCC title.

## Introduction

A study by Weber Shandwick in 2020 found that reputation accounts for 63% of a company’s market value on average. This raises a natural question: why is reputation so important for firms? According to the signal theory, reputation can serve as a signal to alleviate information asymmetry and communicate the firm’s ability to the stakeholders^1^. Prior literature documents that firms with better reputation can obtain higher market value, better financial performance and more innovation outputs^2–4^. In contrast, reputational damage leads to customer loss, revenue decline or even bankruptcy^5,6^. Recognizing the importance of reputation, many firms engage in strategic behaviors to improve their reputation, such as corporate social responsibility activities and charitable donations.

Moreover, firms not only have their own reputations, but also share collective reputation with other firms in the same groups. As a important type of collective reputation, city reputation is the perception of a place by potential investors, businesses, and workers^7,8^. It also reflects the attractiveness and competitiveness of local firms. For example, Silicon Valley in San Francisco is widely recognized as a global center for innovation and technology. The reputation of this city benefits local firms, as it attracts more talent, investment, and collaboration opportunities^9,10^. Despite the great value creation of city reputation for firms, the literature has largely ignored its impact on corporate behavior. In this paper, we use the staggered adoption of the National Civilized City (NCC) policy in China as a natural experiment to examine the impact of city reputation on corporate risk-taking. The NCC award is the highest honor for the civilization level of cities, and many governments pursue it to improve their image and reputation^11–13^. Thus, this policy provides an exogenous shock that enhances city reputation. We focus on risk-taking because it is crucial to corporate survival and success^14,15^.

Based on the above analyses, this paper selects the public firms traded on the Chinese A-share market over the period 2001 to 2018 as our sample. We first investigate the relationship between city reputation and corporate risk-taking. Second, we shed light on the channels through which city reputation affects corporate risk-taking. Finally, we examine how information asymmetry moderates the impact of city reputation on corporate risk-taking.

Our paper contributes to the literature in the following ways. First, city reputation is a valuable asset for local economy, but measuring it is challenging. Existing research has used the number of tourists^16^ or surveys of people’s impression of cities^7,8^ as a proxy for city reputation. However, these measures may not be objective or independent of local economic conditions. This paper exploits the staggered implementation of the NCC policy as a natural experiment to improve city reputation^11–13^. Therefore, our paper provides a reliable and valid method to evaluate the real effect of city reputation.

Second, this paper expands the literature on the effects of city reputation on micro-firms. Prior studies have focused on the impact of city reputation on economic performance^7,8^, air pollution^12^ and energy efficiency^11^. However, the implications for corporate business have been largely overlooked. The only related study is by Zhao et al.^13^, who explore the relationship between city reputation and stock price crash risk. Different from this paper, we further enrich the literature by shedding light on the impact of city reputation on corporate risk-taking.

Finally, this paper also broadens the research on how regional culture influences corporate risk-taking. Berry-Stölzle and Irlbeck^17^ find that firms located in more religious regions tends to obtain lower risk-taking. Huang et al.^18^ study the clan culture in China and claim that higher clan culture can limit corporate risk-taking behavior. Shen et al.^19^ demonstrate that social trust has a significant role in decreasing corporate risk-taking. Khieu et al.^20^ also suggest that corruption culture can deter firms from making risky investments, resulting in lower risk-taking. Unlike these papers, we show that city reputation, a key aspect of regional culture, can act as an informal mechanism to encourage firms to take more risks.

## Institutional background

Despite the rapid economic growth in recent decades, China confronts several challenges such as environmental pollution and human health problems^21,22^. To address these issues, China starts a campaign to improve the quality of urban development. In 1996, the Chinese government proposed to build national civilization cities in the sixth plenary session of the 14th CPC Central Committee. In 1997, the China Central Civilization Commission was established to implement this project and developed the National Civilized City Evaluation System in 2004. Next, it announced the first batch of NCC in 2005 and the subsequent batches in 2009, 2011, 2015, 2017. As of 2018, the NCC title has been awarded to 122 prefecture-level cities and 54 counties.

According to the website of China Civilization, the NCC award is the highest honor for the civilization level of cities. It reflects the comprehensive achievements of the awarded city in economic, political, cultural, social, ecological, and other aspects. Actually, the list of NCC is updated every three years since 2005. During this period, each province can nominate a group of cities and counties for the NCC award. Then, the China Central Civilization Commission evaluates the scores for these nominees through material review, questionnaire survey, on-site inspection, and report listening. Finally, the new NCC is selected by the China Central Civilization Commission based on the average scores over three-year overlapping period.

In addition, the China Central Civilization Commission issued Dynamic Management Measures for the National Civilized City in 2015. It sets specific penalties for cities that fail to meet the standards after receiving the NCC award, such as deducting evaluation scores, facing public criticism or losing the honor title. These measures motivate the awarded cities and nominated cities to maintain their reputation and improve their performance.

## Hypothesis development

Prior literature offers opposing views on the impact of city reputation on corporate risk-taking. On the one hand, we argue that city reputation can increase corporate risk-taking based on the *benefit hypothesis*. First, city reputation provides firms with easier resource availability, more investments and better financial performance^7,23–25^. These benefits could alleviate corporate resources constraints and improve financial condition. Second, city reputation can create a form of insurance that prevent more damage when firms experience negative events^26,27^. In this case, we suppose that firm may obtain lower default risk. Since better financial condition and lower default risk could encourage firms to take risky projects^28–30^, the *benefit hypothesis* implies that city reputation leads to higher risk-taking. Therefore, we propose the following hypothesis:

### H1a

City reputation is positively associated with corporate risk-taking.

On the other hand, city reputation may lower corporate risk-taking according to the *burden hypothesis*. Zavyalova et al.^31^ argue that high reputation attracts more stakeholder attention. Mishina et al.^32^ and Petkova et al.^33^ mention that high reputation can raise stakeholders’ expectations about future performance of organizations. Therefore, firms located in the NCC may have less incentive to take risks because they face more market penalties in case of negative events^34,35^. Taken together, the *burden hypothesis* expects that city reputation is negatively associated with corporate risk-taking. Our hypothesis is as follows:

### H1b

City reputation is negatively associated with corporate risk-taking.

## Research design

### Data

We collect financial data on public firms traded on the A-share market from the China Stock Market & Accounting Research (CSMAR) Database. The data on media coverage, firm location and city-level economic condition come from the Chinese Research Data Services (CNRDS) database. Our sample starts from 2000 because it is the first year that the CNRDS database provides information on firm location. Our primary sample covers the period from 2001 to 2020. The dependent variable is measured by using annual data over three-year overlapping period, our final sample period thus ends in 2018. Since the control variables are lagged by one year in the empirical model, our sample period spans from 2001 to 2018. We then exclude (1) firms in financial industry and (2) firms that are special treatment (ST) and particular transfer (PT). The final sample consists of 2,912 firms and 30,573 firm-year observations.

### Measuring corporate risk-taking

Following the literature^36–38^, we employ two measures to estimate corporate risk-taking. The first is the volatility of industry-adjusted ROA. We construct *RiskI* as the standard deviation of the 2-digit SIC industry-adjusted ROA (*ROAI*) over the window *t* to *t* + *2*. Therefore, we estimate *RiskI* as follows:

1 $$RiskI_{it} = \sqrt {\frac{1}{N - 1}\mathop \sum \limits_{n = 1}^{N} \left( {ROAI_{in} - \frac{1}{N}\mathop \sum \limits_{n = 1}^{N} ROAI_{in} } \right)^{2} } /N = 3$$

The second proxy for risk-taking is the maximum difference value of adjusted ROA. Specifically, *RiskIM* equals the maximum minus the minimum adjusted ROA over three overlapping years. *RiskIM* is defined as follows:

2 $$RiskIM_{it} = Max\left( {ROAI_{in} } \right) - Min\left( {ROAI_{in} } \right)/N = 3$$

### Measuring city reputation

Following Li et al.^11^, Liu et al.^12^ and Zhao et al.^13^, we use whether a city/county is selected as the NCC to measure city reputation. We obtain the data on NCC from the website of China Civilization, published by the China Central Civilization Commission since 2005. Specifically, if a city/county where the firm’s headquarter is located has won the NCC title, Treat*Post equals one and zero otherwise.

### Empirical model

We employ staggered DID approach to investigate the relationship between city reputation and corporate risk-taking. The model is as follows:

3 $$Risk_{i,t} = \alpha + \beta *Treat_{i} *Post_{t - 1} + \gamma *CONTROLS_{i,t - 1} + \delta_{i} + \delta_{t} + \varepsilon_{i,t}$$

where ${Risk}_{i,t}$ is corporate risk-taking of firm *i* in year *t*, which is measured by *RiskI* and *RiskIM*. $Treat_{i}$ equals one if the city/county where firm *i* is located has ever adopted the NCC policy. $Post_{t - 1}$ equals one if the city/county where firm *i* is located is selected as the NCC in year *t−1* and zero otherwise. We use the coefficient $\beta$ to capture the effect of city reputation on corporate risk-taking. Following the literature^38–40^, $CONTROLS_{i,t - 1}$ includes firm size (*Size*), firm leverage (*Lev*), sales growth (*Growth*), return on asset (*ROA*), market-to-book ratio (*MB*), firm age (*Age*), capital expenditures (*Capex*) and the growth rate of GDP (*GDPgrowth*). Detailed definitions of these variables are provided in Table 1. $\delta_{i}$ and $\delta_{t}$ denote firm and year fixed effects, respectively*.* The continuous variables are winsorized at the 1% and 99% levels. Finally, we cluster standard errors at the city-year level.

**Table 1:** Variable definitions.

Variable	Data description
*RiskI*	The volatility of industry-adjusted ROA over the window *t* to *t* + *2*
*RiskIM*	The maximum minus the minimum adjusted ROA the window *t* to *t* + *2*
*RiskA*	The volatility of ROA over the window *t* to *t* + *2*
*RiskAM*	The maximum minus the minimum ROA the window *t* to *t* + *2*
Treat*Post	An indicator variable that equals one if a city that the firm is located wins the NCC award in year *t*, and zero otherwise
Treat*PostA	An indicator variable that equals one for firms located in its prefecture-level city if a county wins the NCC award, and zero otherwise
Treat*PostR	An indicator variable that equals one if firms are assigned with the treated status in the random sample in year *t*, and zero otherwise
*COCAP*	The average of γ_PEG_ calculated in the PEG model over the window *t* to *t* + *2*
*COCAM*	The average of γ_MPEG_ calculated in the MPEG model over the window *t* to *t* + *2*
*ZScoreA*	The average of ZScore calculated in Eq. (8) over the window *t* to* t* + *2*
*LevA*	The average of the ratio of total debt scaled by total assets over the window *t* to *t* + *2*
*WCA*	The average of the ratio of working capital scaled by total assets over the window *t* to *t* + *2*
*Size*	The logarithm of total output
*Lev*	The ratio of total debt to total assets
*Growth*	The growth rate of total sales
*ROA*	The net profit divided by total assets
*MB*	The ratio of market value to total assets
*Age*	The logarithm of the number of years since the firm was founded
*Capex*	The ratio of capital expenditures to total assets
*GDPgrowth*	The GDP growth rate in the city
*Board*	The number of directors in log amount
*Indep*	The ratio of independent directors
*Dual*	An indicator variable that equals one if the chairman and CEO are the same person and zero otherwise

### Descriptive statistics

Table 2 reports summary statistics of the main variables. For the dependent variables, *RiskI* and *RiskIM* have means at 0.073 and 0.135, with the standard deviations of 0.234 and 0.412 respectively. The mean of Treat*Post is 0.383, suggesting that 38.3% of firms are located in cities with the NCC award in our sample. The average values of *Size*, *Lev*, *Growth*, *ROA*, *MB*, *Age*, *Capex* are 21.735, 0.450, 0.223, 0.036, 1.974, 2.648 and 0.056 respectively. Moreover, we find that a prefecture-level city has an average GDP growth rate of 10.4% during our sample period.

**Table 2:** Descriptive statistics.

	N	MEAN	STD	P25	P50	P75
*RiskI*	30,573	0.073	0.234	0.015	0.025	0.048
*RiskIM*	30,573	0.135	0.412	0.029	0.048	0.092
Treat*Post	30,573	0.383	0.486	0.000	0.000	1.000
*Size*	30,573	21.735	1.254	20.835	21.577	22.431
*Lev*	30,573	0.450	0.212	0.286	0.448	0.607
*Growth*	30,573	0.223	0.536	*− *0.009	0.132	0.312
*ROA*	30,573	0.036	0.058	0.013	0.036	0.064
*MB*	30,573	1.974	1.263	1.223	1.556	2.215
*Age*	30,573	2.648	0.432	2.398	2.708	2.944
*Capex*	30,573	0.056	0.055	0.016	0.039	0.078
*GDPgrowth*	30,573	0.104	0.141	0.085	0.119	0.169

## City reputation and corporate risk-taking

### Main result

To examine how city reputation affects corporate risk-taking, we employ a staggered DID approach based on Eq. (3). Panel A of Table 3 shows the results. We find that the coefficient on Treat*Post in column (1) is significant at 0.0220 (*t value* = 4.16). This finding suggests that corporate risk-taking significantly increases after firms are located in the NCC. In terms of economic impact, the NCC award is associated with a 2.20% increase in risk-taking, which is equivalent to 30.1% (= 0.0220/0.073) of the mean of *RiskI.* This result is consistent with the *benefit hypothesis*: city reputation can improve financial condition and reduce default risk, leading to a higher level of risk-taking tolerance. We find a similar result in column (2) when we use *RiskIM* to measure the dependent variable.

**Table 3:** City reputation and corporate risk-taking.

Dependent variable	RiskI	RiskIM
	(1)	(2)
**Panel A: baseline results**		
Treat*Post	0.0220***	0.0384***
	(4.16)	(4.14)
*Size*	*− *0.0155***	*− *0.0271***
	(*− *3.95)	(*− *3.92)
*Lev*	0.0832***	0.1499***
	(4.76)	(4.88)
*Growth*	*− *0.0012	*− *0.0026
	(*− *0.35)	(*− *0.41)
*ROA*	*− *0.2619***	− 0.4813***
	(− 5.18)	(− 5.41)
*MB*	0.0020	0.0037
	(0.98)	(1.00)
*Age*	− 0.0388**	− 0.0653**
	(− 2.50)	(− 2.40)
*Capex*	− 0.0876**	− 0.1567**
	(− 2.40)	(− 2.44)
*GDPgrowth*	0.0312**	0.0514**
	(2.30)	(2.13)
Firm fixed effects	Yes	Yes
Year fixed effects	Yes	Yes
N	30,573	30,573
R^2^	0.282	0.286
**Panel B: stacked DID estimates**		
Treat*Post	0.0443**	0.0757**
	(2.21)	(2.07)
*Size*	− 0.0777***	− 0.1379***
	(− 3.38)	(− 3.34)
*Lev*	0.2276**	0.4031**
	(2.31)	(2.26)
*Growth*	0.0014	0.0020
	(0.13)	(0.10)
*ROA*	− 0.7100**	− 1.3216**
	(− 2.10)	(− 2.14)
*MB*	− 0.0193**	− 0.0341**
	(− 2.36)	(− 2.31)
*Age*	− 0.1681**	− 0.2974**
	(− 2.17)	(− 2.15)
*Capex*	0.0024	− 0.0140
	(0.01)	(− 0.03)
*GDPgrowth*	0.2352**	0.4257**
	(2.47)	(2.46)
Firm-cohort fixed effects	Yes	Yes
Year-cohort fixed effects	Yes	Yes
N	660,058	660,058
R^2^	0.355	0.354

Significance is indicated at the 10% (*), 5% (**), and 1% (***) levels.

### Stacked DID estimates

Recent studies show that there is potential bias related to staggered DID estimates in the existence of “bad comparisons” problem^41–44^. Following Gormley and Matsa^45^ and Cengiz et al.^46^, we employ a stacked DID approach to address this issue. Specifically, we construct a cohort set (a specific dataset) for each city receiving the NCC award. Each cohort incorporates observations in a [*− *5,5] window ranging from 5 years before the NCC policy adoption to 5 years after the year. Then, we keep cities without the NCC title within this period to get clean control groups. Finally, we append all cohort sets into a total sample and conduct the following model.

4 $$Risk_{i,c,t} = \alpha + \beta *Treat_{i,c} *Post_{t - 1} + \gamma *CONTROLS_{i,c,t - 1} + \delta_{i,c} + \delta_{c,t} + \varepsilon_{i,c,t}$$

where $Risk_{i,c,t}$ is corporate risk-taking in firm *i*, cohort *c* and year *t*. The definitions of $Treat_{i,c} *Post_{t - 1}$ and $CONTROLS_{i,c,t}$ follow Eq. (3). Firm-cohort fixed effects $\delta_{i,c}$ and year-cohort fixed effects $\delta_{c,t}$ are included in Eq. (3). We also cluster standard errors at the city-year level. As shown in Panel B of Table 3, we find corporate risk-taking still significantly increases in the stacked panel. This finding indicates that our main result is not driven by late treatment bias.

### Robustness checks

#### Alternative measures of corporate risk-taking

Following Boubakri et al.^39^ and Khaw et al.^47^, we use the volatility of ROA over three overlapping years to measure the dependent variable. Specifically, we use ROA to replace *ROAI* in both model (1) and model (2), and then calculate corporate risk-taking, which are denoted as *RiskA* and *RiskAM*. Panel A of Table 4 reports the results of alternative measure of corporate risk-taking. In columns (1) and (2), we find that the coefficients on Treat*Post are both significant at 0.0044 (*t value* = 3.19) and 0.0083 (*t value* = 3.20), consistent with the results in Panel A of Table 3. This finding suggests that city reputation still plays a significant role in increasing corporate risk-taking after considering alternative measures of the dependent variable.

**Table 4:** Robustness checks.

Dependent variable	RiskA	RiskAM		
	(1)	(2)		
**Panel A: alternative measure of corporate risk-taking**				
Treat*Post	0.0044***	0.0083***		
	(3.19)	(3.20)		
*Size*	0.0031***	0.0056***		
	(2.88)	(2.78)		
*Lev*	0.0273***	0.0520***		
	(5.29)	(5.43)		
*Growth*	− 0.0018*	− 0.0034*		
	(− 1.77)	(− 1.84)		
*ROA*	− 0.1656***	− 0.3074***		
	(− 11.69)	(− 11.73)		
*MB*	0.0026***	0.0048***		
	(4.54)	(4.43)		
*Age*	0.0131***	0.0243***		
	(3.31)	(3.34)		
*Capex*	− 0.0524***	− 0.0962***		
	(− 6.12)	(− 6.06)		
*GDPgrowth*	− 0.0014	− 0.0033		
	(− 0.25)	(− 0.32)		
Firm fixed effects	Yes	Yes		
Year fixed effects	Yes	Yes		
N	30,573	30,573		
R^2^	0.415	0.417		
**Panel B: alternative measure of city reputation**				
Treat*PostA	0.0212***	0.0373***		
	(3.88)	(3.87)		
*Size*	− 0.0154***	− 0.0268***		
	(− 3.92)	(− 3.89)		
*Lev*	0.0834***	0.1503***		
	(4.78)	(4.90)		
*Growth*	− 0.0013	− 0.0026		
	(− 0.36)	(− 0.42)		
*ROA*	− 0.2625***	− 0.4824***		
	(− 5.19)	(− 5.42)		
*MB*	0.0021	0.0038		
	(0.99)	(1.02)		
*Age*	− 0.0377**	− 0.0634**		
	(− 2.43)	(− 2.34)		
*Capex*	− 0.0884**	− 0.1580**		
	(− 2.42)	(− 2.47)		
*GDPgrowth*	0.0329**	0.0545**		
	(2.42)	(2.26)		
Firm fixed effects	Yes	Yes		
Year fixed effects	Yes	Yes		
N	30,573	30,573		
R^2^	0.282	0.286		

Dependent variable	RiskI	RiskIM	RiskI	RiskIM
	(1)	(2)	(3)	(4)
**Panel C: alternative samples**				
Treat*Post	0.0217***	0.0378***	0.0189***	0.0329***
	(4.07)	(4.05)	(2.72)	(2.68)
*Size*	− 0.0157***	− 0.0274***	− 0.0131**	− 0.0225**
	(− 3.98)	(− 3.94)	(− 2.56)	(− 2.50)
*Lev*	0.0844***	0.1520***	0.0880***	0.1592***
	(4.82)	(4.93)	(3.77)	(3.85)
*Growth*	− 0.0012	− 0.0025	− 0.0013	− 0.0023
	(− 0.34)	(− 0.41)	(− 0.25)	(− 0.24)
*ROA*	− 0.2616***	− 0.4809***	− 0.1720**	− 0.3280***
	(− 5.15)	(− 5.38)	(− 2.52)	(− 2.71)
*MB*	0.0021	0.0039	0.0015	0.0025
	(1.03)	(1.06)	(0.61)	(0.58)
*Age*	− 0.0376**	− 0.0633**	− 0.0329	− 0.0540
	(− 2.42)	(− 2.33)	(− 1.56)	(− 1.46)
*Capex*	− 0.0845**	− 0.1513**	− 0.0351	− 0.0636
	(− 2.30)	(− 2.35)	(− 0.74)	(− 0.76)
*GDPgrowth*	0.0314**	0.0519**	0.0436***	0.0745**
	(2.30)	(2.14)	(2.65)	(2.54)
Firm fixed effects	Yes	Yes	Yes	Yes
Year fixed effects	Yes	Yes	Yes	Yes
N	30,403	30,403	15,704	15,704
R^2^	0.282	0.285	0.300	0.304

Significance is indicated at the 10% (*), 5% (**), and 1% (***) levels.

#### Alternative measure of city reputation

According to the list of NCC, many counties participate in competition for the NCC award with prefecture-level cities. It is possible that the city can obtain high reputation when its county wins the NCC title. For example, the county Zhangjiagang was selected as the NCC in 2005, we suppose that its prefecture-level city Suzhou would have an increase in city reputation. We then use this alternative variable Treat*PostA and re-estimate Eq. (3). Panel B of Table 4 shows that the coefficients on Treat*PostA in two columns are both positive and significant at 1% level. Accordingly, these findings indicate that our main result is not driven by alternative measure of city reputation.

#### Alternative samples

In our sample, firm characteristics and regional economic conditions are matched at the prefecture-level cities. Therefore, we control for city-level characteristics even for those firms located in counties with the NCC title. One might worry that our main result is driven by counties that won the NCC award earlier than their prefecture-level cities. Following Zhao et al.^13^, we then exclude these observations to ensure robust estimation. Panel C of Table 4 reports the results. In columns (1) and (2), our main inference remains the same after addressing this issue.

To mitigate the concern of regional differences, we exclude firms located in provincial capitals and four municipalities (Beijing, Shanghai, Tianjin and Chongqing). The results are shown in Panel C of Table 4. Columns (3) and (4) show that the association between city reputation and corporate risk-taking is still positive and significant in the restricted sample. These results suggest that our main result is not sensitive to regional differences.

## Identification issues

### Test of parallel trends assumption

The validity of our main result in Eq. (4) is based on the parallel trends assumption. That is, the treatment and control groups should exhibit comparable corporate risk-taking trends before the NCC policy is implemented. To test this assumption, we follow Beck et al.^48^ and examine the dynamic effect of city reputation on corporate risk-taking based on the following model.

5 $${Risk}_{i,t}=\alpha +{\beta }_{1}*{Pre 6+}_{i,t-1}+{\beta }_{2}*{Pre 5}_{i,t-1}+{\beta }_{3}*{Pre 4}_{i,t-1}+{\beta }_{4}*{Pre 3}_{i,t-1}+{\beta }_{5}*{Pre 2}_{i,t-1}+{\beta }_{6}*{Current}_{i,t-1}+{\beta }_{6}*{Post 1}_{i,t-1}+{\beta }_{6}*{Post 2}_{i,t-1}+{\beta }_{6}*{Post 3}_{i,t-1}+{\beta }_{6}*{Post 4}_{i,t-1}+{\beta }_{6}*{Post 5}_{i,t-1}+{\beta }_{6}*{Post 6+}_{i,t-1}+\gamma *{CONTROLS}_{i,t-1}+{\delta }_{i}+{\delta }_{t}+{\varepsilon }_{i,t}$$

We use the following dummy variables to indicate the timing of the NCC award for firms located in prefecture-level cities: *Pre 6* + (six or more years before the award), Pre *j* (*j* = 2, 3, 4, 5 years before the award), *Current* (the year of the award), *Post 1* (one year after the award), *Post j* (*j* = 2, 3, 4, 5 years after the award), and *Post 6* + (six or more years after the award). We assign a value of one to the corresponding variable and zero otherwise. The other variables are the same as in Eq. (3).

**Figure 1:**
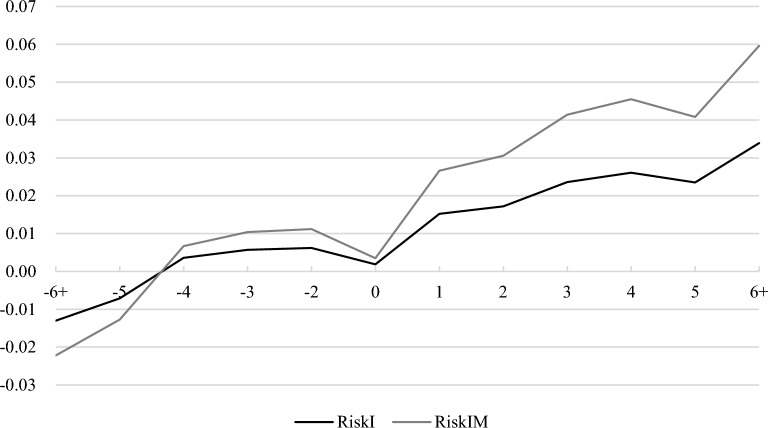
The dynamic effect of city reputation.

We present the results in Panel A of Table 5. In column (1), we find that *Pre 6* + , *Pre 5*, *Pre 4*, *Pre 3*, *Pre 2* play an insignificant role in explaining the increase in *RiskI*. This finding supports the parallel trends assumption by showing no systematic difference in corporate risk-taking between firms located in NCC and non-NCC. Moreover, column (1) shows that the effect of city reputation emerges one year after gaining the NCC award. Using *RiskIM* as the dependent variable, our result in column (2) is consistent with column (1). Finally, we plot the regression coefficient estimates in Fig. 1 based on the [*− *6 + , 6 +] window. Figure 1 shows that our main result is not driven by pre-event period, which further verifies parallel trends assumption.

**Table 5:** Identification issues.

Dependent variable	RiskI	RiskIM	
	(1)	(2)	
**Panel A: testing the parallel trends assumption**			
*Pre 6* +	− 0.0130	− 0.0222	
	(− 1.42)	(− 1.37)	
*Pre 5*	− 0.0071	− 0.0127	
	(− 0.70)	(− 0.71)	
*Pre 4*	0.0036	0.0067	
	(0.31)	(0.33)	
*Pre 3*	0.0057	0.0104	
	(0.63)	(0.66)	
*Pre 2*	0.0062	0.0112	
	(0.66)	(0.68)	
*Current*	0.0019	0.0035	
	(0.20)	(0.22)	
*Post 1*	0.0152*	0.0266*	
	(1.73)	(1.72)	
*Post 2*	0.0172*	0.0306*	
	(1.66)	(1.67)	
*Post 3*	0.0236***	0.0414***	
	(2.58)	(2.58)	
*Post 4*	0.0261**	0.0455**	
	(2.42)	(2.42)	
*Post 5*	0.0235***	0.0408***	
	(2.85)	(2.81)	
*Post 6* +	0.0339***	0.0596***	
	(3.64)	(3.63)	
*Size*	− 0.0156***	− 0.0272***	
	(− 3.97)	(− 3.93)	
*Lev*	0.0821***	0.1480***	
	(4.71)	(4.83)	
*Growth*	− 0.0012	− 0.0026	
	(− 0.35)	(− 0.42)	
*ROA*	− 0.2621***	− 0.4816***	
	(− 5.18)	(− 5.41)	
*MB*	0.0022	0.0040	
	(1.05)	(1.07)	
*Age*	− 0.0389**	− 0.0655**	
	(− 2.48)	(− 2.38)	
*Capex*	− 0.0877**	− 0.1568**	
	(− 2.40)	(− 2.45)	
*GDPgrowth*	0.0307**	0.0505**	
	(2.29)	(2.12)	
Firm fixed effects	Yes	Yes	
Year fixed effects	Yes	Yes	
N	30,573	30,573	
R^2^	0.282	0.286	
**Panel B: Placebo test**			
Treat*PostR	0.0005	0.0009	
	(0.21)	(0.19)	
*Size*	− 0.0151***	− 0.0264***	
	(− 3.85)	(− 3.82)	
*Lev*	0.0822***	0.1482***	
	(4.71)	(4.83)	
*Growth*	− 0.0015	− 0.0029	
	(− 0.41)	(− 0.48)	
*ROA*	− 0.2632***	− 0.4835***	
	(− 5.20)	(− 5.43)	
*MB*	0.0021	0.0039	
	(1.02)	(1.05)	
*Age*	− 0.0410***	− 0.0693**	
	(− 2.65)	(− 2.55)	
*Capex*	− 0.0875**	− 0.1565**	
	(− 2.40)	(− 2.44)	
*GDPgrowth*	0.0295**	0.0484**	
	(2.21)	(2.04)	
Firm fixed effects	Yes	Yes	
Year fixed effects	Yes	Yes	
N	30,573	30,573	
R^2^	0.281	0.285	

Dependent variable	1st stage	2nd stage	2nd stage
	Treat*Post	RiskI	RiskIM
	(1)	(2)	(3)
**Panel C: instrumental variable approach**			
*IV*	0.0001***		
	(4.18)		
Treat*Post		0.1642**	0.3060**
		(2.42)	(2.44)
*Size*	0.0045	0.0108***	0.0198***
	(0.63)	(5.29)	(5.28)
*Lev*	− 0.0803***	0.0302***	0.0570***
	(− 3.27)	(3.38)	(3.46)
*Growth*	− 0.0015	− 0.0011	− 0.0022
	(− 0.34)	(− 0.87)	(− 0.96)
*ROA*	0.0733	− 0.0601***	− 0.1063***
	(1.15)	(− 3.20)	(− 3.07)
*MB*	0.0082***	0.0013	0.0024
	(3.44)	(1.46)	(1.47)
*Age*	0.4093***	− 0.0171	− 0.0366
	(6.91)	(− 0.51)	(− 0.60)
*Capex*	− 0.0408	− 0.0237	− 0.0414
	(− 0.71)	(− 1.42)	(− 1.34)
*GDPgrowth*	0.0140	0.0370*	0.0672*
	(0.19)	(1.77)	(1.74)
Firm fixed effects	Yes	Yes	Yes
Year fixed effects	Yes	Yes	Yes
N	15,470	15,470	15,470
R^2^	0.008	− 0.508	− 0.528
Cragg-Donald Wald F statistic		17.440	17.440

Significance is indicated at the 10% (*), 5% (**), and 1% (***) levels.

### Placebo test

Another endogeneity issue is that our main result could be driven by a linear combination of the independent variable. To address this concern, we follow Mao and Zhang^49^ and conduct placebo test. In our sample, there are 38.3% firms located in cities with NCC award. We then randomly select 38.3% firms from the full sample as the false treatment groups. Treat*PostR equals one if a firm is assigned to treatment status in the random sample and zero otherwise. We re-estimate Eq. (3) and present the results in Panel B of Table 5. In columns (1) and (2), we find that city reputation is not significantly associated with corporate risk-taking. This finding suggests that our main inference is not an artifact of the data structure.

### Instrumental variable approach

Finally, we use instrumental variable approach to further address the omitted variable concern. Specifically, we use the number of scenic spots (*IV*) as the instrumental variable. On the one hand, this variable is highly correlated with city reputation. More scenic spots tend to attract a greater number of tourists from other regions, leading to a higher city reputation. On the other hand, scenic spots often are influenced by a city’s history and geography, which is exogenous to corporate risk-taking. Then, we collect scenic spots information from the CNRDS database, which is available after 2011. Panel C of Table 5 reports the result of instrumental variable approach. In column (1), we find the coefficient on *IV* is positive and significant at 1% level. This finding is consistent with our prediction that cities with more scenic spots tend to obtain better reputation. The results in column (2) and (3) show that city reputation significantly increases corporate risk-taking.

## Channel tests

Our main result suggests that firms located in the NCC have better financial condition and lower default risk, leading to a higher level of risk-taking. In this section, we first test the impact of city reputation on cost of capital. Second, we investigate the relationship between city reputation and default risk. Finally, we examine how information asymmetry moderates the relationship between city reputation and corporate risk-taking.

### Cost of capital

First, we use cost of capital to measure corporate financial condition. Koirala et al.^30^ claim that lower cost of capital may encourage firms to engage in more risk-taking activities. We then suppose that firms located in cities with the NCC title are more likely to exhibit cheaper equity financing. To test this conjecture, we employ the PEG and MPEG approach proposed by Easton^50^ to estimate the cost of capital, denoted as $\gamma_{PEG}$ and $\gamma_{MPEG}$. Parallel to our risk-taking proxies, we follow Ferris et al.^51^ and calculate the cost of capital as the average of $\gamma_{PEG}$ and $\gamma_{MPEG}$ over the window *t* to *t* + *2*. As shown in Panel A of Table 6, we find the coefficients on Treat*Post are significant at *− *0.0021 (*t value* = *− *2.61) and *− *0.0036 (*t value* = *− *3.19) in columns (1) and (2), indicating that firms headquartered in NCC obtain lower cost of capital than firms in non-NCC. This finding confirms our prediction that city reputation can bring firms with better financial condition and thus increase corporate risk-taking.

**Table 6:** Channel tests.

Dependent variable	COCAP	COCAM	ZScoreA	
	(1)	(2)	(3)	
**Panel A: The channel of financial condition**				
Treat*Post	− 0.0021***	− 0.0036***	− 0.0016**	
	(− 2.61)	(− 3.19)	(− 2.55)	
*Size*	0.0031***	0.0051***	− 0.0096***	
	(4.42)	(5.29)	(− 16.28)	
*Lev*	0.0027	− 0.0138***	− 0.0525***	
	(0.99)	(− 3.64)	(− 24.36)	
*Growth*	0.0019***	0.0013*	0.0006	
	(3.46)	(1.94)	(1.43)	
*ROA*	0.0151**	0.0356***	0.0303***	
	(1.99)	(3.09)	(5.32)	
*MB*	0.0010***	0.0004	0.0042***	
	(4.07)	(1.07)	(9.61)	
*Age*	− 0.0018	− 0.0064	− 0.0025*	
	(− 0.47)	(− 1.29)	(− 1.66)	
*Capex*	0.0138***	0.0016	− 0.0322***	
	(2.73)	(0.25)	(− 8.77)	
*GDPgrowth*	0.0028	− 0.0019	0.0011	
	(1.32)	(− 0.58)	(0.56)	
Firm fixed effects	Yes	Yes	Yes	
Year fixed effects	Yes	Yes	Yes	
N	11,016	11,016	29,799	
R2	0.720	0.719	0.743	

Dependent variable	Less analyst following		More analyst following	
	RiskI	RiskIM	RiskI	RiskIM
	(1)	(2)	(3)	(4)
**Panel B: The moderating role of analyst following**				
Treat*Post	0.0320***	0.0565***	0.0088	0.0149
	(4.02)	(4.04)	(1.36)	(1.31)
*Size*	− 0.0141*	− 0.0251*	− 0.0149**	− 0.0244**
	(− 1.93)	(− 1.96)	(− 2.22)	(− 2.07)
*Lev*	0.1072***	0.1927***	− 0.0055	− 0.0084
	(4.26)	(4.36)	(− 0.21)	(− 0.18)
*Growth*	− 0.0070	− 0.0128	0.0135**	0.0231**
	(− 1.38)	(− 1.44)	(2.38)	(2.33)
*ROA*	− 0.3631***	− 0.6607***	0.0491	0.0728
	(− 5.16)	(− 5.33)	(0.70)	(0.58)
*MB*	− 0.0032	− 0.0060	0.0032	0.0057
	(− 0.83)	(− 0.88)	(1.52)	(1.58)
*Age*	− 0.0131	− 0.0217	− 0.1005**	− 0.1740**
	(− 0.59)	(− 0.56)	(− 2.49)	(− 2.47)
*Capex*	− 0.0659	− 0.1203	− 0.1083*	− 0.1876*
	(− 1.14)	(− 1.19)	(− 1.92)	(− 1.90)
*GDPgrowth*	0.0499**	0.0833**	0.0391***	0.0674***
	(2.19)	(2.06)	(2.67)	(2.61)
Firm fixed effects	Yes	Yes	Yes	Yes
Year fixed effects	Yes	Yes	Yes	Yes
N	15,119	15,119	14,645	14,645
R^2^	0.365	0.369	0.326	0.328

Dependent variable	Less media coverage		More media coverage	
	RiskI	RiskIM	RiskI	RiskIM
	(1)	(2)	(3)	(4)
**Panel C: The moderating role of media coverage**				
Treat*Post	0.0312***	0.0552***	0.0131	0.0230
	(3.55)	(3.56)	(1.43)	(1.43)
*Size*	− 0.0107	− 0.0187	− 0.0263***	− 0.0463***
	(− 1.35)	(− 1.34)	(− 4.24)	(− 4.24)
*Lev*	0.0775***	0.1377***	0.0599**	0.1079**
	(2.58)	(2.60)	(2.26)	(2.30)
*Growth*	− 0.0087	− 0.0154	0.0087*	0.0149*
	(− 1.52)	(− 1.52)	(1.71)	(1.67)
*ROA*	− 0.2838***	− 0.5190***	− 0.2449***	− 0.4581***
	(− 3.69)	(− 3.82)	(− 3.36)	(− 3.54)
*MB*	0.0013	0.0021	0.0014	0.0026
	(0.34)	(0.30)	(0.45)	(0.46)
*Age*	− 0.0493*	− 0.0830*	− 0.0836***	− 0.1440***
	(− 1.79)	(− 1.72)	(− 3.04)	(− 2.98)
*Capex*	− 0.0609	− 0.1087	− 0.0778	− 0.1371
	(− 1.02)	(− 1.04)	(− 1.35)	(− 1.35)
*GDPgrowth*	0.0446*	0.0749	0.0419*	0.0703*
	(1.71)	(1.62)	(1.80)	(1.69)
Firm fixed effects	Yes	Yes	Yes	Yes
Year fixed effects	Yes	Yes	Yes	Yes
N	13,707	13,707	12,359	12,359
R^2^	0.364	0.368	0.367	0.370

Dependent variable	Less corporate replies		More corporate replies	
	RiskI	RiskIM	RiskI	RiskIM
	(1)	(2)	(3)	(4)
**Panel D: the moderating role of investor interaction**				
Treat*Post	0.0089**	0.0158**	− 0.0006	− 0.0005
	(2.38)	(2.33)	(− 0.17)	(− 0.09)
*Size*	0.0188***	0.0347***	0.0234***	0.0438***
	(4.64)	(4.67)	(6.39)	(6.53)
*Lev*	0.0128	0.0244	− 0.0019	− 0.0016
	(0.74)	(0.77)	(− 0.15)	(− 0.07)
*Growth*	− 0.0038*	− 0.0075*	− 0.0001	− 0.0007
	(− 1.69)	(− 1.84)	(− 0.06)	(− 0.14)
*ROA*	0.0220	0.0500	− 0.0789**	− 0.1406**
	(0.67)	(0.83)	(− 2.56)	(− 2.50)
*MB*	0.0027**	0.0049**	0.0016	0.0028
	(2.04)	(2.00)	(1.54)	(1.52)
*Age*	0.0825**	0.1493**	0.0711**	0.1316**
	(2.40)	(2.37)	(2.51)	(2.54)
*Capex*	− 0.0770***	− 0.1374***	− 0.0183	− 0.0341
	(− 2.76)	(− 2.70)	(− 0.66)	(− 0.67)
*GDPgrowth*	0.0037	0.0055	0.0044	0.0073
	(0.53)	(0.43)	(0.62)	(0.55)
Firm fixed effects	Yes	Yes	Yes	Yes
Year fixed effects	Yes	Yes	Yes	Yes
N	7,921	7,921	7,813	7,813
R^2^	0.610	0.612	0.550	0.553

Significance is indicated at the 10% (*), 5% (**), and 1% (***) levels.

### Default risk

Second, we examine how city reputation affects default risk. Hilscher and Raviv^28^ and Favara et al.^29^ document that firms with higher default risk adopt less risk-taking activities. Theoretically, if obtaining the NCC title can provide firms with a form of insurance, we predict that it may reduce the likelihood of default risk. To examine this hypothesis, we follow Altman^52^ and use Z-Score to capture default risk. We define *ZScoreA* as the average of *ZScore* over three-year overlapping period. We report the result in Panel A of Table 6. This result in column (3) indicates that firms located in the NCC are indeed more likely to obtain lower default risk. As predicted, these results suggest that city reputation could improve financial condition and reduce default risk, and thus motivate firms to take more risk-taking behaviors.

### Heterogeneous effects

In this section, we examine the moderating role of information asymmetry. Li et al.^26^ argue that country reputation may have weaker effect when investors have access to more specific information from other sources. Since information asymmetry could strengthen the power of reputation, we expect that the positive relationship between city reputation and corporate risk-taking is pronounced for firms with higher information asymmetry.

To test this prediction, we use three measures to capture the information asymmetry: analyst following, media coverage and investor interaction. Roulstone^53^ and Frankel and Li^54^ find that analyst following can improve the informativeness between managers and investors. As such, we suppose that city reputation has a stronger effect on increasing risk-taking for firms with less analyst following. We first calculate analyst following as the log of (1 + the number of analysts). The data on analyst following come from the CSMAR database during 2001 to 2018. Then, our sample is divided into two groups according to the median of analyst following. Panel B of Table 6 shows the results. In columns (1) and (2), we find that city reputation demonstrates a significantly positive association with corporate risk-taking. In contrast, the coefficients on Treat*Post are both insignificant in columns (3) and (4). These results suggest that the impact of city reputation on corporate risk-taking is more pronounced for firms with less analyst following, which is consistent with our prediction.

Second, we investigate the moderating effect of media coverage. Prior studies document that the media can simplify and explain complex information for market participants, alleviating the mispricing of accounting information^55–57^. We thus expect the effect of city reputation to be stronger for firms with less media coverage. Following You et al.^58^, we select eight leading newspapers to capture media coverage. We collect newspaper data from the CNRDS database, spanning the period 2001 to 2018. A firm is viewed as having higher (less) information asymmetry if its media coverage of “Big 8” is lower (greater) than the median of all firms. We then run separate regressions for the groups and present the results in Panel C of Table 6. We find that city reputation exhibits a significant role in risk-taking for firms with less media coverage, but not for more media coverage.

Finally, we test whether the impact of city reputation on corporate risk-taking varies across firms with different investor interaction. Blankespoor^59^ and Lee and Zhong^60^ suggest that investor interactive platform may significantly reduce information processing costs and improve corporate transparency. In this case, increasing investor interaction may weaken the effect of city reputation on risk-taking tolerance. Following Lee and Zhong^60^, we use the number of replies posted by the firm on the investor interactive platform to measure information asymmetry. The more replies by target firms, the less information asymmetry. The information on investor interaction comes from the CNRDS database over the period 2010 to 2018. We then divide our sample into two groups based on the sample median. As shown in Panel D of Table 6, the coefficients on Treat*Post are positive and significant in columns (1) and (2) while insignificant in columns (3) and (4).

## Additional analyses

### The effect of corporate governance

Prior research documents that better corporate governance can encourage firms to pursue riskier activities, leading to a higher corporate risk-taking^30,61,62^. It is possible that the increasing risk-taking has been attributed to the improvement of corporate governance with the passage of the NCC policy. To exclude this explanation, we follow Deutsch et al.^62^ and include the number of directors in log amount (*Board*), the ratio of independent directors (*Indep*), and CEO–chairman duality (*Dual*) in our baseline model. We gather corporate governance data from the CSMAR database, covering the period from 2001 to 2018. We re-estimate Eq. (3) and present the results in Panel A of Table 7. In columns (1) and (2), we find that the role of city reputation in explaining corporate risk-taking is still significant after controlling for corporate governance. These results suggest that our results are not driven by a higher corporate governance associated with higher city reputation.

**Table 7:** Additional analyses.

Dependent variable	RiskI	RiskIM		
	(1)	(2)		
**Panel A: the effect of corporate goverance**				
Treat*Post	0.0104***	0.0190***		
	(2.63)	(2.63)		
*Size*	− 0.0104***	− 0.0189***		
	(− 2.96)	(− 2.96)		
*Lev*	0.0783***	0.1456***		
	(5.26)	(5.40)		
*Growth*	0.0019	0.0029		
	(0.67)	(0.57)		
*ROA*	− 0.1698***	− 0.3138***		
	(− 4.46)	(− 4.54)		
*MB*	0.0011	0.0019		
	(0.80)	(0.75)		
*Age*	0.0279	0.0523		
	(1.49)	(1.55)		
*Capex*	− 0.0885***	− 0.1631***		
	(− 3.04)	(− 3.08)		
*Board*	0.0280**	0.0513**		
	(2.51)	(2.54)		
*Indep*	0.0057	0.0150		
	(0.18)	(0.27)		
*Dual*	0.0003	0.0004		
	(0.07)	(0.05)		
*GDPgrowth*	0.0149	0.0251		
	(1.60)	(1.48)		
Firm fixed effects	Yes	Yes		
Year fixed effects	Yes	Yes		
N	25,798	25,798		
R^2^	0.316	0.318		

Dependent variable	LevA	WCA		
	(1)	(2)		
**Panel B: city reputation and corporate financial policies**				
Treat*Post	0.0084***	− 0.0060**		
	(3.71)	(− 2.16)		
*Size*	0.0186***	− 0.0135***		
	(10.78)	(− 6.09)		
*Lev*	0.5180***	− 0.3973***		
	(56.82)	(− 36.60)		
*Growth*	− 0.0001	0.0079***		
	(− 0.04)	(3.99)		
*ROA*	− 0.2337***	0.4585***		
	(− 11.57)	(17.85)		
*MB*	0.0010	− 0.0025**		
	(1.06)	(− 1.97)		
*Age*	0.0261***	− 0.0964***		
	(3.68)	(− 11.08)		
*Capex*	0.1289***	− 0.3956***		
	(8.48)	(− 20.30)		
*GDPgrowth*	− 0.0027	− 0.0010		
	(− 0.37)	(− 0.11)		
Firm fixed effects	Yes	Yes		
Year fixed effects	Yes	Yes		
N	30,573	30,573		
R^2^	0.822	0.777		

Dependent variable	Industries with high competition		Industries with low competition	
	RiskI	RiskIM	RiskI	RiskIM
	(1)	(2)	(3)	(4)
**Panel C: industry characteristics**				
Treat*Post	0.0430***	0.0757***	0.0048	0.0078
	(4.07)	(4.12)	(1.28)	(1.17)
*Size*	− 0.0341***	− 0.0593***	0.0034	0.0061
	(− 4.70)	(− 4.68)	(0.98)	(0.98)
*Lev*	0.1445***	0.2543***	0.0452***	0.0866***
	(4.31)	(4.34)	(3.03)	(3.20)
*Growth*	0.0035	0.0055	− 0.0050	− 0.0088
	(0.66)	(0.59)	(− 1.64)	(− 1.58)
*ROA*	− 0.2236**	− 0.4196**	− 0.2505***	− 0.4567***
	(− 2.32)	(− 2.49)	(− 5.63)	(− 5.71)
*MB*	− 0.0042	− 0.0066	0.0046**	0.0077**
	(− 1.02)	(− 0.91)	(2.27)	(2.14)
*Age*	− 0.1344***	− 0.2314***	0.0029	0.0066
	(− 4.01)	(− 3.96)	(0.24)	(0.31)
*Capex*	− 0.1408*	− 0.2552**	− 0.0199	− 0.0333
	(− 1.93)	(− 2.01)	(− 0.75)	(− 0.69)
*GDPgrowth*	0.1260***	0.2152***	− 0.0080	− 0.0165
	(3.39)	(3.31)	(− 0.85)	(− 0.96)
Firm fixed effects	Yes	Yes	Yes	Yes
Year fixed effects	Yes	Yes	Yes	Yes
N	12,642	12,642	17,931	17,931
R^2^	0.290	0.293	0.323	0.329

Significance is indicated at the 10% (*), 5% (**), and 1% (***) levels.

### City reputation and corporate financial policies

Next, we examine whether firms located in NCC adopt riskier financial policies. Following Ferris et al.^51^, we employ two measures of financial policies. The first is the leverage ratio, which reflects the proportion of debt in the capital structure. The second is the working capital ratio, which proxies for the liquidity of the firm. We estimate these two measures by averaging them over a three-year period from year *t* to year *t* + *2*, denoted as *LevA* and *WCA* respectively. As shown in Panel B of Table 7, in column (1), Treat*Post shows a significantly positive association with *LevA*, indicating that firm leverage significantly increases after the adoption of the NCC policy. We also find that city reputation plays a significant role in decreasing working capital in column (2). These results indicate that better city reputation could motivate firms to adopt riskier financial policies by increasing more debt burden and holding less liquid assets.

### Industry characteristics

Finally, we investigate the impact of city reputation on corporate risk-taking across different levels of industrial competition. On the one hand, high competition often drives firms to invest more to stay competitive, which may decrease financial resources^63,64^. On the other hand, increased competition can also pose more risks to firms, leading to a greater default risk. We thus suppose that the positive relationship between city reputation and corporate risk-taking is more pronounced for firms with high industrial competition level. Following Jiang et al.^64^, we use the Herfindahl–Hirschman Index (HHI) to capture the industry’s competition. An industry is classified as high (low) competition if its HHI exceeds (falls below) the median across all industries.. Panel C of Table 7 reports the results, indicating that the treated firms in highly competitive industries tend to engage in more risk-taking.

## Conclusion and discussion

### Conclusion

In this paper, we examine how city reputation affects corporate risk-taking. Utilizing the staggered adoption of the NCC policy in China, our DID analysis finds that city reputation exhibits a significant role in increasing corporate risk-taking. To address endogeneity issues, we first test the parallel trends assumption. We find that no divergent trend emerges in corporate risk-taking prior to receiving the NCC award. Second, we conduct the placebo test. The results show that our main result is not an artifact of the data structure. Finally, our main inference still holds after using instrumental variable approach to address the omitted variable concern.

In the channel tests, we first examine whether city reputation may motivate firms to adopt risk-taking behavior through improving financial condition and reducing default risk. The results suggest that the treated firms experience a significant decrease in the cost of capital and default risk. Then, we conduct cross-section tests to investigate the moderating role of information asymmetry. We find that the positive relationship between city reputation and corporate risk-taking is more pronounced for firms with less analyst following, less media coverage and less corporate replies. In additional analyses, we confirm that firms located in the NCC pursue riskier financial policies, including more debt burden and less liquid assets. We also find that the impact of city reputation on corporate risk-taking is more pronounced in highly competitive industries.

Overall, this paper sheds light on the important role of city reputation in shaping corporate risk-taking behavior. Our results also indicate that city reputation may be a informal mechanism to alleviate financial constraints and stimulate value-enhancing investment. This paper provides empirical evidence for local governments to pursue and maintain a better city reputation.

### Policy implications

First, the government should recognize the value creation of city reputation and adopt relevant policies to enhance it. In the current economy, city reputation indicates the competitiveness and quality of the local economy. For example, we associate San Francisco with technology, London with finance, and Detroit with automobiles. In this case, city reputation can act as a signal to reduce information asymmetry and attract more capital and human capital. This is in line with our finding, which shows that better city reputation encourages firms to undertake more risky investments and increases corporate risk-taking tolerance. Meanwhile, the government should also emphasize the externality of reputation and prevent activities that may damage city reputation.

Second, firms should recognize the important role of city reputation for improving financial performance. Due to underdeveloped financial market, 75% of firms in China suffer from financial constraints, particularly for private and small enterprises^65^. Although the Chinese government has made great efforts to ease financing difficulties, the problem caused by financial constraints remains a significant obstacle for firm development and investment. Our finding suggests that city reputation can decrease cost of capital and default risk. Thus, firms should strive to build and maintain a good reputation to alleviate financial constraint and increase financial stability.

Finally, it is clear that information asymmetry leads to market failure and inefficient resource allocation^66^. However, our finding reveals that city reputation has a stronger positive effect on corporate risk-taking for firms with higher information asymmetry. This suggests that city reputation can act as an information channel to help investors evaluate firm attributes. Therefore, the government should enhance city reputation to reduce information frictions and improve corporate governance.

### Limitations

First, our study only covers the public firms listed on the Chinese A-share market. These firms may have more access to financial resources and lower financial risk. Thus, our results may understate the effect of city reputation on corporate risk-taking. In fact, private firms contribute the most to GDP and employment in China^67^. However, these firms face severe financial constraints and high financial costs. Therefore, future research can explore how city reputation influences corporate behavior in these firms.

Second, city reputation is a complex concept that captures how a city is viewed by its residents, visitors, investors, and other stakeholders. Measuring this indicator is challenging and difficult. Although our paper uses the NCC title, the highest honor for the civilization level of cities, as a proxy for city reputation, it may not fully reflect all aspects of city reputation.

Finally, we follow the previous literature and use corporate risk-taking to capture a firm's propensity towards engaging in risky activities^36–38^. However, it’s worth noting that this dependent variable primarily measures the scale of effect rather than the probability of risk. Recent studies have shed light on more detailed and precise methodologies for assessing the likelihood scale of risk^68–70^. We will pay close attention to this field in the future.

## Data Availability

The datasets used of this study is available from the corresponding author on reasonable request.
